# Public Awareness and Knowledge of Sleeve Gastrectomy in the Southwest Region of Saudi Arabia

**DOI:** 10.7759/cureus.64344

**Published:** 2024-07-11

**Authors:** Omar A Alshaikhi, Mohamed E Salih, Afnan H Awadh, Khadejah K Sindi, Atheer N Alkenani, Rahaf M Alsaedi, Mohammed A Aljidaani, Abdullah A Alzubaidi, Mohannad A Alshaikhi, Mohammed Himmat, Hassan A AlZubaidi, Saleh A Alshaikhi

**Affiliations:** 1 Pediatrics, Ministry of Health, Jeddah, SAU; 2 Department of Surgery, Al-Qunfudah Medical College, Umm Al-Qura University, Al-Qunfudah, SAU; 3 General and Colorectal Surgery, Wad Medani Hospital, Medani, SDN; 4 Department of Medicine and Surgery, Al-Qunfudah Medical College, Umm Al-Qura University, Al-Qunfudah, SAU; 5 Department of Medicine and Surgery, Hayatt University College, Khartoum, SDN; 6 Department of Family and Community Medicine, King Abdulaziz Medical City, National Guard Health Affairs, Jeddah, SAU; 7 Family Medicine, Ministry of Health, Jeddah, SAU

**Keywords:** southwest region, bmi, complications, indications, bariatric surgery, obesity, sleeve gastrectomy

## Abstract

Background

Obesity is a major global health concern, causing significant health dilemmas. Large groups of Saudi individuals are considered obese, with significant implications for medical practice. Bariatric surgery, including sleeve gastrectomy, is a crucial intervention for severe obesity, although it is associated with potential complications. This study aims to investigate the public knowledge about the indications and consequences of sleeve gastrectomy in the Southwest region of Saudi Arabia and assess their general awareness of sleeve gastrectomy.

Methodology

This descriptive, cross-sectional, online-based study included 347 individuals from the Southwest region of Saudi Arabia. Data were collected via an online questionnaire and analyzed using SPSS version 27 (IBM Corp., Armonk, NY, USA).

Results

This study included 347 participants, with a majority being females (88.5%, n = 307) and Saudis (98.6%, n = 342). The most common age group was 21-25 years (34%, n = 118), followed by those over 40 years of age (26.5%, n = 92). Most participants resided in Al-Qunfudhah (66.3%, n = 230) and held a bachelor’s degree (75.8%, n = 263). Nearly half were students (48.7%, n = 169), and 56.2% (n = 195) earned less than 5,000 per month. The prevalent weight range was 40-60 kg (46.1%, n = 160), and most participants’ height was 150-160 cm (58.5%, n = 203). Overall, 61% (x̄ = 211.6) of the respondents had good knowledge about sleeve gastrectomy, with 70.3% (x̄ = 244) understanding its general aspects, 56.1% (x̄ = 194.5) knowing the indications, and 60.1% (x̄ = 208.7) aware of the complications.

Conclusions

The majority of residents of the Southwest region of Saudi Arabia have moderate levels of knowledge regarding sleeve gastrectomy. However, the study demonstrated substantial gaps in knowledge and awareness regarding sleeve gastrectomy, mainly about its indications and potential.

## Introduction

Obesity has emerged as a significant global health challenge in recent years [1], constituting the most prevalent metabolic disorder attaining epidemic proportions across both developed and developing nations [2,3]. As of 2016, approximately 13% of the global adult population was affected by obesity, with rates of 15% among women and 11% among men [4].

In Saudi Arabia, obesity is one of the major health concerns affecting both genders, with more than 35% of the population being obese [5,6]. As a result, several affected individuals seek metabolic bariatric surgery (MBS) without previous knowledge of the indications and its consequences. As they can be drawn and influenced by other people or media advertisements, it is important for them to understand and be aware of this topic and its indications.

Addressing obesity necessitates a systematic approach. Initially, all patients should prioritize lifestyle modifications and behavioral therapy. Pharmacological interventions represent the second line of management. MBS constitutes the third-line treatment, acknowledged as a highly efficacious and enduring intervention, particularly for severely to moderately obese individuals who show inadequate response to non-surgical modalities [3]. Apart from sustainable weight loss, MBS demonstrates optimal glycemic control, leading to diabetes remission [7].

Specific guidelines for MBS must be meticulously observed, which vary by procedure type [8,9], with sleeve gastrectomy emerging as the predominant procedure. Sleeve gastrectomy is efficient not only due to its restrictive nature but also because of the removal of the gastric fundus which leads to lower ghrelin levels, reduced appetite, and weight loss [10,11]. Despite its efficacy, this procedure carries potential complications, some of which are life-threatening [12]. Major complications include leaks and hemorrhages [13]. Postoperative complications, ranging from 4.4% to 12.8%, encompass a spectrum, including anastomotic leaks, hemorrhages, abscesses, nutritional deficiencies, venous thromboses, surgical site infections, and other rare occurrences [14-16].

In Saudi Arabia, indications for sleeve gastrectomy encompass adults with a body mass index (BMI) of 30 kg/m² or greater, particularly those with poorly controlled type 2 diabetes and heightened cardiovascular risk, adults with a BMI of 35 kg/m² or greater presenting severe comorbidities, or adults with a BMI of 40 kg/m² or greater [17,18].

Although various studies have explored the landscape of obesity and sleeve gastrectomy across Saudi Arabia, to date, no investigation has focused specifically on the Southwest region. Thus, the objective of this study is to enhance our comprehension of public awareness and knowledge regarding sleeve gastrectomy in the Southwest region of Saudi Arabia. A recent nationwide study conducted by Taha et al. (2024) examined the awareness among Saudi Arabian residents about the consequences of sleeve gastrectomy. This study included 1,013 participants, predominantly aged between 18 and 25 years (471, 46%) and predominantly female (692, 68%). A notable majority, 987 (97%) participants, demonstrated awareness of gastric sleeve surgery, with 538 (53%) accurately understanding its indications. Concerning complications associated with sleeve gastrectomy, approximately 821 (81%) participants exhibited awareness. The study also revealed significant correlations between the knowledge of gastric sleeve surgery and residence in the northern region of Saudi Arabia [19].

Numerous studies have explored public awareness and understanding of sleeve gastrectomy across various regions of Saudi Arabia. For instance, Buhalim et al. conducted a study in 2023 focusing on the Eastern Province, involving 1,730 participants predominantly aged 18-25, predominantly female, and with a bachelor’s degree. Despite high awareness of sleeve gastrectomy (99%), only 50.1% accurately identified the BMI range for obesity classification, and knowledge scores indicated varying levels of understanding (61.7% poor, 31% moderate, 7.2% good). Correct identification of sleeve gastrectomy indications stood at 56.1%, while awareness of complications, including nutritional deficiencies, was notable but incomplete [20].

Similarly, Abdulrahman et al. conducted a study in Riyadh in 2023 involving 1,700 respondents, predominantly female (67.4%) and with a bachelor’s degree (64.0%). Awareness of sleeve gastrectomy complications was recognized by 43.9%, although 33.1% were unaware of associated risks, indicating a generally positive attitude toward the procedure [21].

Alolayan et al. conducted another study in 2021 in Al-Qassim involving 1,091 respondents, who were predominantly female (87.8%) with a mean age of 37.5 years. Awareness rates were 49% for indications and 82.4% for complications, with knowledge significantly associated with awareness of BMI. The study found high awareness of sleeve gastrectomy (99.1%) among the participants, with 60.9% aware of indications and 72.2% of complications. Knowledge levels were higher among those aware of BMI [22].

Lastly, Al Watban et al. explored awareness among 480 participants in Riyadh in 2020, with a majority male (51.5%) and educated up to bachelor’s level (52.7%). While awareness of BMI was limited (16.7% knew the correct obese BMI levels), awareness of sleeve gastrectomy complications was higher (64.8%), correlating with educational level [23].

Therefore, this study aims to fill the research gap in the understanding of the public awareness and knowledge of sleeve gastrectomy in the Southwest region of Saudi Arabia, as, to our knowledge, no studies have been conducted in this region to assess this concern.

## Materials and methods

Study design

A descriptive, cross-sectional, online-based study was conducted in the Southwest region of Saudi Arabia by utilizing a structured self-administered online questionnaire targeting the general population in the region.

Study objective

The objective of this study was to assess the level of public knowledge about the indications and consequences of sleeve gastrectomy in the Southwest region of Saudi Arabia and assess their general awareness of sleeve gastrectomy.

Inclusive criteria

Male and female adults, aged 18 years and more, living in the Southwest region of Saudi Arabia who accepted to participate in this study were included.

Sample size

Sampling was done based on the convenient sampling method with an expected sample size of 347 according to Raosoft calculation taking into consideration a 5% margin of error and a 95% confidence interval.

Data collection tools

The data were collected through an online questionnaire using e-mails and other social media platforms including WhatsApp, X, and Telegram. A cover page that illustrated both the purpose of the study and participant consent was used. The questionnaire included four sections. The first one included the sociodemographic characteristics of the participants such as gender, age, body weight, and height. The second section included five questions to assess the general knowledge of participants. The third section contained seven questions to assess the knowledge regarding the indications of sleeve gastrectomy. The fourth section included nine questions to assess the knowledge of participants regarding the complications of sleeve gastrectomy.

Data analysis

After obtaining consent to conduct the study, the data were collected and computerized using Microsoft Excel (Microsoft Corp., Redmond, WA, USA) to form a database using all the items from the data collection sheets. Data were then encoded and analyzed using SPSS version 27 (IBM Corp., Armonk, NY, USA). Data are displayed and presented in the form of tables and charts.

Ethical considerations

Ethical approval was sought from Umm Al-Qura University (approval number: HAPO-02-K-012-2024-02-2021) before starting the study. The objectives and benefits of the study were explained to the participants. Confidentiality and privacy of the participants were maintained. The participants had the right to withdraw consent at any time without any consequences.

## Results

A total of 347 participants were included in this study. There were more females (88.5%, n = 307) than males (11.5%, n = 40). The most common age group was 21-25 years (34%, n = 118), followed by more than 40 years (26.5%, n = 92). The majority (98.6%, n = 342) were Saudi, while (1.4%, n = 5) were non-Saudi. The most common place of residence was Al-Qunfudhah (66.3%, n = 230), followed by Al-Qouz (10.4%, n = 36). The majority (75.8%, n = 263) had a bachelor’s degree, followed by a secondary school degree (15.6%, n = 54), Almost half of the respondents (48.7%, n = 169) were students, followed by employees (32.3%, n = 112). Meanwhile, the majority (56.2%, n = 195) earned less than 5,000 per month, followed by 10,000-15,000 (18.7%, n = 65) (Table 1).

**Table 1:** Demographic distribution.

Variables		Frequency	Percent	Valid percent	Cumulative percent
Gender	Male	40	11.5	11.5	11.5
	Female	307	88.5	88.5	100.0
Age (years)	15–20	58	16.7	16.7	16.7
	21–25	118	34.0	34.0	50.7
	26–30	26	7.5	7.5	58.2
	31–35	22	6.3	6.3	64.5
	36–40	31	8.9	8.9	73.5
	More than 40	92	26.5	26.5	100.0
Nationality	Saudi	342	98.6	98.6	98.6
	Non-Saudi	5	1.4	1.4	100.0
Residency	Al-Qunfudhah	230	66.3	66.3	66.3
	Al-Qouz	36	10.4	10.4	76.7
	Almuzaylif	11	3.2	3.2	79.9
	Jeddah	6	1.7	1.7	81.6
	Hali	28	8.1	8.1	89.7
	Makkah	15	4.3	4.3	94.0
	Other	21	6.0	6.0	100.0
Education	None	4	1.2	1.2	1.2
	Secondary school	54	15.6	15.6	16.8
	High school	3	0.8	0.8	17.6
	Bachelor’s degree	263	75.8	75.8	93.4
	Master degree	16	4.6	4.6	98.0
	P.H. degree	7	2.0	2.0	100.0
Occupation	Student	169	48.7	48.7	48.7
	Employee	112	32.3	32.3	81.0
	Unemployed	40	11.5	11.5	92.5
	Retired	26	7.5	7.5	100.0
Monthly income	Less than 5,000	195	56.2	56.2	56.2
	5,000–10,000	43	12.4	12.4	68.6
	10,000–15,000	65	18.7	18.7	87.3
	15,000–20,000	29	8.4	8.4	95.7
	More than 20,000	15	4.3	4.3	100.0
Total		347	100.0	100.0	100.0

Almost half of the respondents (46.1%, n = 160) weighed 40-60 kg, followed by 60-80 kg (30%, n = 104). The majority (58.5%, n = 203) had a height of 150-160 cm, followed by 160-170 (24.5%, n = 85) (Table 2).

**Table 2:** Biometric distribution.

Variables	Category	Count	Percentage
Weight (kg)	Less than 40	36	10.4%
	40–60	160	46.1%
	60–80	104	30%
	80–100	41	11.8%
	100–120	5	1.4%
	More than 120	1	0.3%
Height (cm)	Less than 150	38	11%
	150–160	203	58.5%
	160–170	85	24.5%
	170–180	19	5.5%
	180–190	2	0.6%
Total		347	100%

The overall good knowledge of respondents regarding sleeve gastrectomy was found to be 61% (x̄ = 211.6), with 70.3% (x̄ = 244) having good general knowledge about sleeve gastrectomy, 56.1% (x̄ = 194.5) having good knowledge about indications of sleeve gastrectomy, and 60.1% (x̄ = 208.7) having good knowledge about complications of sleeve gastrectomy (Tables 3, 4).

**Table 3:** Responses of participants regarding sleeve gastrectomy.

Variables	Category	Count	Percentage
General knowledge about sleeve gastrectomy			
Did you hear about sleeve gastrectomy?	Yes	337	97.1%
	No	10	2.9%
What is the BMI range that we can say this person is obese? (kg/m^2^)	Less than 18.5	8	2.3%
	18.5–24.9	43	12.4%
	25–29.9	52	15%
	More than 30	140	40.3
	I don’t know	104	30%
Do you know about the indication of sleeve gastrectomy?	Yes	249	71.8%
	No	98	28.2%
Do you know about the complications of sleeve gastrectomy?	Yes	250	72%
	No	97	28%
Knowledge about indications of sleeve gastrectomy			
An adult with a BMI >40 is an indication of sleeve gastrectomy	Yes	189	54.5%
	No	44	12.7%
	I don’t know	114	32.9%
An adult with a BMI >35 and severe comorbidities is an indication of sleeve gastrectomy	Yes	190	54.8%
	No	48	13.8%
	I don’t know	109	31.4%
An adult with BMI >30, poorly controlled type 2 diabetes, increased cardiovascular risk is an indication for sleeve gastrectomy	Yes	183	52.7%
	No	47	13.5%
	I don’t know	117	33.7%
An adult with a BMI from 18.5 to 24.9 is an indication of sleeve gastrectomy	Yes	46	13.3%
	No	187	53.9%
	I don’t know	114	32.9%
An adult with a BMI of 18.5 is an indication of sleeve gastrectomy	Yes	36	10.4%
	No	200	57.6%
	I don’t know	111	32%
An adult with a BMI less than 18.5 is an indication of sleeve gastrectomy	Yes	30	8.6%
	No	218	62.8%
	I don’t know	99	28.5%
Knowledge about complications of sleeve gastrectomy			
Hemorrhage is a possible complication of sleeve gastrectomy	Yes	241	69.5%
	No	21	6.0%
	I don’t know	85	24.5%
A leak of gastric content is a possible complication of sleeve gastrectomy	Yes	241	69.5%
	No	18	5.1%
	I don’t know	88	25.4%
Abscess is a possible complication of sleeve gastrectomy	Yes	182	52.4%
	No	27	7.8%
	I don’t know	138	39.8%
Iron deficiency is a possible complication of sleeve gastrectomy	Yes	258	74.4%
	No	18	5.1%
	I don’t know	71	20.5%
Anemia is a possible complication of sleeve gastrectomy	Yes	235	67.7%
	No	31	8.9%
	I don’t know	81	23.3%
Twisting of the stomach is a possible complication of sleeve gastrectomy	Yes	176	50.7%
	No	27	7.8%
	I don’t know	144	41.5%
Pulmonary emboli is a possible complication of sleeve gastrectomy	Yes	150	43.2%
	No	38	11%
	I don’t know	159	45.8%
Neuropathies is a possible complication of sleeve gastrectomy	Yes	148	42.7%
	No	38	11%
	I don’t know	161	46.3%
Weight regain is a possible complication of sleeve gastrectomy	Yes	247	71.2%
	No	24	6.9%
	I don’t know	76	21.9%
Total		347	100%

**Table 4:** Overall knowledge of respondents regarding sleeve gastrectomy. x̄: statistical mean

Variables	Mean (x̄)	Percentage
General knowledge about sleeve gastrectomy	244	70.3%
Knowledge about indications of sleeve gastrectomy	194.5	56.1%
Knowledge about complications of sleeve gastrectomy	208.7	60.1%
Overall knowledge about sleeve gastrectomy	211.6	61%

## Discussion

This study aimed to assess the knowledge and awareness of sleeve gastrectomy indications and complications among residents of the Southwest region of Saudi Arabia. Several studies conducted across different regions of Saudi Arabia have examined similar themes, contributing valuable insights to the existing literature.

Our study found that the overall level of knowledge among respondents regarding sleeve gastrectomy was moderate. Specifically, a substantial proportion exhibited good general knowledge about sleeve gastrectomy, with a majority showing familiarity with its indications and potential complications. These findings underscore the importance of education and awareness initiatives to further enhance understanding among the general population regarding this surgical procedure.

A recent study by Taha et al. involving a substantial sample of 1,013 participants reported a high awareness rate of sleeve gastrectomy (97%) among Saudi Arabian residents. The majority of participants demonstrated awareness of BMI (68%), and a significant proportion were familiar with the complications associated with sleeve gastrectomy (81%). However, only 53% of participants accurately understood the indications for sleeve gastrectomy [19].

Similarly, Buhalim et al., in a study involving 1,730 participants, highlighted widespread awareness of sleeve gastrectomy (99%). Despite this high awareness, only 56.1% of respondents correctly identified the indications for undergoing sleeve gastrectomy. The study also revealed varying levels of knowledge, with 61.7% demonstrating poor knowledge, 31% moderate knowledge, and 7.2% good knowledge levels regarding sleeve gastrectomy [20].

Abdulrahman et al. conducted a study in Riyadh with 1,700 participants and reported that while a significant proportion of participants were aware of the complications associated with sleeve gastrectomy (43.9%), a notable proportion (33.1%) were unaware of these risks, suggesting potential gaps in public knowledge [21].

Alolayan et al.’s 2021 study in Al-Qassim involved 1,091 respondents and found moderate awareness levels regarding the indications (49%) and high awareness regarding the complications (82.4%) associated with sleeve gastrectomy. These findings closely parallel the results observed in this study, which reported good knowledge levels among 56.1% of respondents regarding indications and 60.1% regarding complications. The study showed nearly universal awareness of sleeve gastrectomy (99.1%) among participants, with 60.9% correctly identifying indications and 72.2% understanding complications. These findings were somewhat higher than those observed in this study, suggesting potential regional or demographic differences [22].

Conversely, Al Watban et al.’s 2020 study in Riyadh revealed lower overall awareness levels and a lack of knowledge about sleeve gastrectomy indications (59.0%) among participants. This study highlighted potential educational disparities and the need for targeted educational interventions [23].

Overall, these studies collectively underscore the ongoing need for comprehensive public education and awareness campaigns regarding sleeve gastrectomy indications and complications across Saudi Arabia. Variations in findings may be attributed to differences in study populations, cultural contexts, and regional demographics, emphasizing the importance of tailored approaches to improve knowledge and understanding among potential candidates and the broader public.

Recommendations

Healthcare providers should work on increasing public awareness about sleeve gastrectomy. Proper counseling should be provided to patients, explaining the indications and possible complications of sleeve gastrectomy. Social media can play a bigger role in raising knowledge and awareness about sleeve gastrectomy. More studies regarding this topic should be conducted to support the evidence and raise knowledge and awareness levels.

Limitations

The study was conducted using a blind methodology on a general population sample without limiting the focus to individuals with specific diseases. Due to the lack of access to bariatric surgery clinics for direct interviews with affected participants, data collection relied on online questionnaires.

## Conclusions

The majority of residents in the Southwest region of Saudi Arabia had moderate levels of knowledge regarding sleeve gastrectomy. However, the study demonstrated substantial gaps in knowledge and awareness regarding sleeve gastrectomy, mainly about its indications and potential complications. It is essential to raise the general public’s knowledge and aware­ness about the indications and complications of sleeve gastrectomy.
